# Alternative Approaches to Bilateral Stellate Ganglion Block for Treatment of Refractory Ventricular Arrhythmia

**DOI:** 10.1155/cria/2691681

**Published:** 2025-12-21

**Authors:** Chelsea Skinner, Mohamad Ayoub, Elie Geara, Adeeb Oweidat, Ismat Mrad, Loran Mounir Soliman, Husien Taleb

**Affiliations:** ^1^ Department of Multispecialty Anesthesiology, Cleveland Clinic, Cleveland, Ohio, USA, clevelandclinic.org; ^2^ Department of Anesthesiology, Perioperative Care & Pain Medicine, NYU Langone Health, New York, New York, USA, nyulangone.org; ^3^ Department of Anesthesia, University of Iowa Healthcare, Iowa City, Iowa, USA; ^4^ Department of Anesthesiology and Perioperative Medicine, University of Rochester Medical Center, Rochester, New York, USA, rochester.edu; ^5^ Scope Anesthesia of North Carolina PLLC, Charlotte, North Carolina, USA

**Keywords:** anesthesia, tachycardia, ultrasound, ventricular tachycardia

## Abstract

**Introduction and Importance:**

Patients with ventricular arrhythmias (VAs) refractory to medical treatment suffer high morbidity and mortality. The stellate ganglion block is an effective diagnostic and therapeutic tool for refractory VAs.

**Case Presentation:**

After obtaining an informed consent, we describe a case of a 62‐year‐old female who suffered ventricular tachycardia refractory to pharmacologic and electrical management, resulting in pulseless ventricular tachycardia (pVT) arrest. The regional anesthesia team was unable to expose the neck and obtain adequate ultrasound visualization using the classic approach and therefore used alternative ultrasound‐guided approaches with success.

**Discussion:**

We describe the performance of the stellate ganglion blocks with an out‐of‐plane needle approach on the left and a medial in‐plane approach on the right. These approaches allowed us to successfully block the stellate ganglia bilaterally.

**Conclusion:**

Refractory VA is associated with an increased cardiac sympathetic tone and has high morbidity and mortality. Stellate ganglion blocks can be used for diagnostic and therapeutic purposes. In‐plane and out‐of‐plane techniques can be utilized.

**Summary**

• To understand the pathophysiology behind refractory VAs and how stellate ganglion blockade can be of diagnostic and therapeutic benefit.
• To explore alternative needle approaches to the ultrasound‐guided stellate ganglion block.
• To understand risks and benefits associated with unilateral (i.e., left‐sided) versus bilateral stellate ganglion blockade.

## 1. Introduction and Importance

Patients with ventricular arrhythmias (VAs) refractory to medical treatment suffer high morbidity and mortality. Refractory VA is associated with an increased sympathetic activity. It can be seen after cardiac surgeries. The stellate ganglion block (SGB), which can be performed by anesthesiologists, is an effective diagnostic and therapeutic tool for refractory VAs. This block has proven effective in reducing the frequency of electrical storms and stabilizing ventricular rhythms in patients where other treatment options have failed [1]. We present a case of refractory VA for which the regional anesthesia service did a SGB. The consent was obtained from the patient’s legal guardian. An alternative approach to the block was performed as the patient was intubated and had a difficult neck access and mobilization.

## 2. Case Presentation

A 62‐year‐old female underwent triple vessel coronary artery bypass grafting two weeks after suffering a missed ST‐segment elevation myocardial infarction that had resulted in pulseless electrical activity arrest. On postoperative Day 3, she suffered ventricular tachycardia refractory to pharmacologic and electrical management, resulting in pulseless ventricular tachycardia (pVT) arrest with subsequent return of spontaneous circulation (ROSC). She required intra‐aortic balloon pump placement at that time. She had multiple subsequent episodes of hemodynamic instability and cardiogenic shock requiring percutaneous left ventricular assist device placement on postoperative Day 5. She also required prolonged mechanical ventilation.

Her medical history was significant for coronary artery disease, heart failure with preserved ejection fraction, hypertension, hyperlipidemia, peripheral vascular disease, chronic obstructive pulmonary disease, and Type II diabetes mellitus.

The differential diagnosis list included supraventricular tachycardia with aberrant conduction, ventricular pre‐excitation syndromes, junctional tachycardia, premature ventricular contractions, myocardial ischemia, and metabolic and/or electrolyte disturbances.

Telemetry monitoring indicated multiple episodes of monomorphic ventricular tachycardia with ventricular rates as high as 220 beats per minute during hospitalization. A bedside transthoracic echocardiogram post‐pVT arrest with ROSC on postoperative Day 3 showed apical, anterior, and septal akinesis and severely reduced ejection fraction. An electrocardiogram at that time revealed no ST‐segment or T wave changes indicative of myocardial ischemia. Subsequent left heart catheterization showed patent bypass grafts.

Given her complicated clinical course, she was deemed prohibitively high‐risk for cardiac ablation. After multidisciplinary discussion, the patient underwent bilateral ultrasound‐guided SGB to stabilize her condition and permit planning for more definitive therapy.

**Figure 1:**
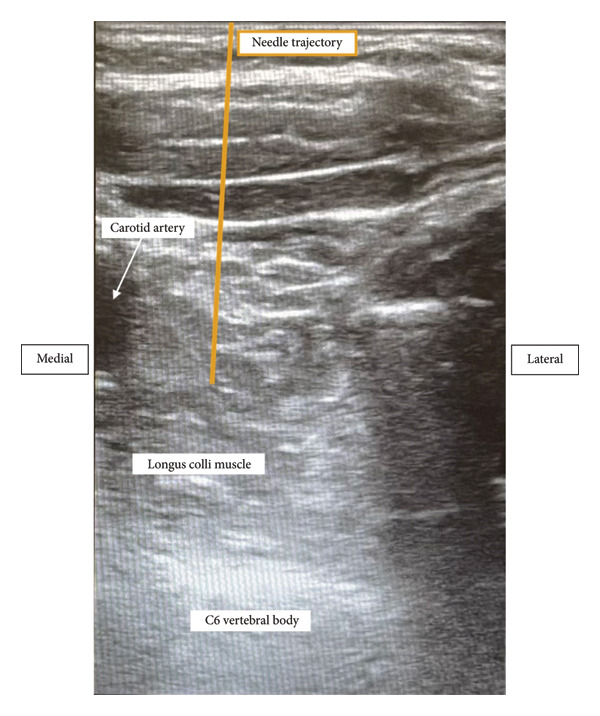
Out‐of‐plane approach to stellate ganglion block. The needle was inserted lateral to the carotid artery and advanced out‐of‐plane (needle trajectory depicted by orange line) to the region of the stellate ganglion anterior to the longus colli muscle.

**Figure 2:**
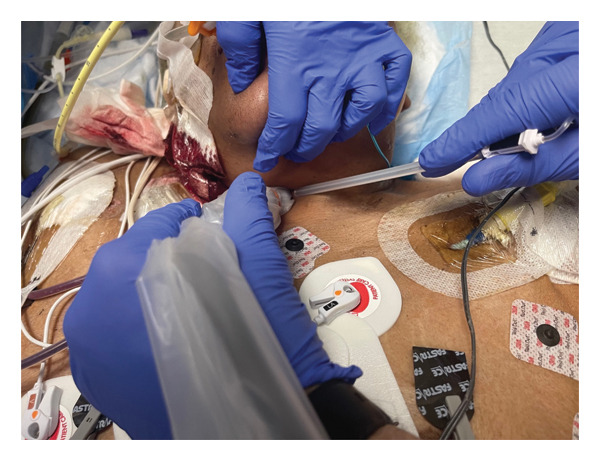
Medial in‐plane approach to stellate ganglion block. Depicted here was our proposed approach to the right‐sided stellate ganglion block. The needle was inserted just lateral to the trachea and advanced in‐plane to the region of the stellate ganglion.

The blocks were performed at bedside in the intensive care unit. The patient was intubated and sedated with very limited neck mobility and bilateral large‐bore central access. It was difficult to obtain adequate visualization and exposure to perform the blocks using the classic ultrasound‐guided approach (i.e., transverse orientation of the probe at the level of the sixth cervical vertebra (C6), inserting the needle lateral to the probe and advancing in‐plane to the stellate ganglion). After thorough ultrasound scanning using a small linear probe, we decided to perform the left SGB with an out‐of‐plane approach. We inserted the needle lateral to the carotid artery and advanced out‐of‐plane to the region of the stellate ganglion (see Figure 1) and injected 7.5 mL of bupivacaine 0.5% and 7.5 mL of lidocaine 1%. Visualization and exposure of the right neck were more challenging since the patient had a right internal jugular sheath introducer in place. We decided to proceed with a medial in‐plane approach by introducing the needle just lateral to the trachea and advancing laterally to reach the stellate ganglion (see Figure 2) and injecting the same local anesthetic mixture. This approach carried the risk of tracheal puncture, and care was taken to keep the needle superficial to the trachea. The patient responded very well to the blocks without further episodes of sustained VA and underwent successful bilateral stellate ganglion neurolysis with ethanol 2 days later.

## 3. Clinical Discussion

Patients with VAs refractory to medical treatment suffer high morbidity and mortality. Furthermore, many of these patients are too critically ill to undergo definitive treatment with ablation of arrhythmogenic foci. The SGB offers a minimally‐invasive therapeutic and diagnostic tool for these patients.

Refractory VA is the result of heightened sympathetic activity. The majority of efferent cardiac sympathetic outflow travels through the stellate ganglion, and it is therefore a target for therapeutic intervention [2]. Surgical cardiac sympathetic denervation (i.e., sympathectomy) is an effective treatment for refractory VA, but surgery is often not an option for unstable patients. Ultrasound‐guided SGB can be performed at bedside and can decrease VA burden and defibrillation events [3]. This block can be used as a temporizing measure in unstable patients as well as a diagnostic tool to identify patients that may benefit from stellate ganglion neurolysis or surgical sympathectomy. Since our patient was deemed prohibitively high‐risk for cardiac ablation, we pursued bedside ultrasound‐guided SGB as temporary therapy and to determine if she would benefit from subsequent sympathectomy or neurolysis. Fortunately, her VA burden drastically decreased, and she underwent successful bilateral stellate ganglion neurolysis 2 days later without recurrence of sustained VA.

The ultrasound‐guided SGB is classically performed with a linear ultrasound probe in transverse orientation at the level of the C6 with the needle inserted lateral to the carotid artery and advanced in‐plane toward the stellate ganglion [4]. This approach minimizes esophageal, thyroid, and vessel injury. Moreover, the performance of this block is more challenging in patients receiving anticoagulation therapy. We were unable to adequately expose our patient’s neck and perform the block in this manner due to her limited neck mobility and bilateral large‐bore central lines. After thorough ultrasound scanning, we opted for an out‐of‐plane needle approach on the left and a medial in‐plane approach on the right. These approaches allowed us to successfully block the stellate ganglia in a safe and ergonomic manner. In addition to that, the modified out‐of‐plane approach shortens the distance to the longus colli muscle, which is beneficial in patients at risk of bleeding [5].

There is debate over whether unilateral or bilateral SGB should be used in patients with VA. A recent meta‐analysis of case reports suggests similar outcomes with the unilateral versus bilateral technique [3]. On the other hand, myocardial injury induces bilateral stellate ganglion remodeling which is reflected in studies that suggest better outcomes with bilateral surgical sympathectomy versus unilateral surgery [6]. Due to the severity of our patient’s presentation, we decided to proceed with bilateral blocks. The SGB carries the risk of temporary recurrent laryngeal and phrenic nerve paralysis. Therefore, simultaneous bilateral blocks should only be performed in the presence of mechanical ventilation. In our case we performed the bilateral SGB while the patient was intubated to prevent any risk of airway compromise. In spontaneously breathing patients, sequential blocks can be performed with a 24 h interval between each procedure.

The remainder of her hospital course was significant for recovery of cardiac function, allowing removal of mechanical circulatory support, dual‐chamber implantable cardioverter defibrillator (ICD) placement, and tracheostomy. She was discharged to a skilled nursing facility, and interrogation of her ICD at a later date showed no evidence of VA.

## 4. Conclusion

Refractory VA is associated with heightened cardiac sympathetic tone and has high morbidity and mortality. Neural modulation via SGB can stabilize these patients enough to pursue more definitive treatment. SGB can also be used as a diagnostic tool to identify patients that may benefit from long‐term neural modulation. The ultrasound‐guided SGB is classically performed with a linear ultrasound probe in transverse orientation at the level of C6 with the needle inserted lateral to the carotid artery and advanced in‐plane toward the stellate ganglion to optimize visualization and minimize injury to surrounding structures. However, if physical constraints prevent the classic approach, an out‐of‐plane approach and a medial in‐plane approach can be safely and effectively utilized. These alternative techniques should be performed only by experienced providers with extensive sonoanatomy knowledge and adequate skills. This will prevent any risks associated with SGB and guarantee safe results. Our patient responded very well to bilateral SGB and subsequent bilateral stellate ganglion neurolysis without recurrence of sustained VA.

**Nomenclature**
C6: Sixth cervical vertebra
ICD: Implantable cardioverter defibrillator
pVT: Pulseless ventricular tachycardia
ROSC: Return of spontaneous circulation
SGB: Stellate ganglion block
VA: Ventricular arrhythmia

## Conflicts of Interest

The authors declare no conflicts of interest.

## Funding

No funding was received for this manuscript.

## Data Availability

Research data are not shared.
